# Progress in molecular feature of smoldering mantle cell lymphoma

**DOI:** 10.1186/s40164-021-00232-3

**Published:** 2021-07-13

**Authors:** Panruo Jiang, Aakash Desai, Haige Ye

**Affiliations:** 1 Department of Hematology, The First Affiliated Hospital of Wenzhou Medical University - Zhejiang, Wenzhou, China; 2 Division of Hematology, Department of Medicine, Mayo Clinic-MN, Rochester, US

**Keywords:** Smoldering mantle cell lymphoma, Indolent, Molecular features

## Abstract

Mantle cell lymphoma (MCL) is considered one of the most aggressive lymphoid tumors. However, it sometimes displays indolent behavior in patients and might not necessitate treatment at diagnosis; this has been described as “smoldering MCL” (SMCL). There are significant differences in the diagnosis, prognosis, molecular mechanisms and treatments of indolent MCL and classical MCL. In this review, we discuss the progress in understanding the molecular mechanism of indolent MCL to provide insights into the genomic nature of this entity. Reported findings of molecular features of indolent MCL include a low Ki-67 index, CD200 positivity, a low frequency of mutations in TP53, a lack of SOX11, normal arrangement and expression of MYC, IGHV mutations, differences from classical MCL by L-MCL16 assays and MCL35 assays, an unmutated P16 status, few defects in ATM, no NOTCH1/2 mutation, Amp 11q gene mutation, no chr9 deletion, microRNA upregulation/downregulation, and low expression of several genes that have been valued in recent years (SPEN, SMARCA4, RANBP2, KMT2C, NSD2, CARD11, FBXW7, BIRC3, KMT2D, CELSR3, TRAF2, MAP3K14, HNRNPH1, Del 9p and/or Del 9q, SP140 and PCDH10). Based on the above molecular characteristics, we may distinguish indolent MCL from classical MCL. If so, indolent MCL will not be overtreated, whereas the treatment of classical MCL will not be delayed.

## Background

Mantle cell lymphoma (MCL) is a mature B-cell neoplasm that accounts for 5% to 10% of all lymphomas [1, 2]. MCL represents a subtype of non-Hodgkin lymphoma with a poor prognosis and is considered to be one of the most aggressive lymphoid tumors [2–5]. However, some display indolent behavior in patients that might not necessitate treatment at diagnosis [3]. In recent years, it has become increasingly clear that MCL is more heterogeneous than we initially thought [6]. In the 2017 WHO Classification of Tumours of Haematopoietic and Lymphoid Tissues, MCL was classified into indolent leukemic non-nodal MCL or classical MCL. Classical MCL is characterized by unmutated/minimally mutated IGHV and mostly SOX11 + , whereas indolent leukemic non-nodal MCL is defined as MCL in which the patient presents with mutated IGHV and mostly SOX11– and peripheral blood, bone marrow, and sometimes splenic involvement but without significant adenopathy. Of note, in situ mantle cell neoplasia, a new name for in situ MCL, is also an indolent type of MCL, reflecting its low clinical risk (Table 1) [7]. However, we found that the significance of IGHV mutations and the expression level of SOX11 in MCL are controversial [8]. In addition, there are many terms used to describe indolent MCL, such as leukemic non-nodal MCL, early-stage MCL, limited-stage MCL, low-risk MCL, and small-MCL [9–15]. After reviewing a large number of previous studies, we recommend that all indolent MCL presentations be unified under the title “smoldering mantle cell lymphoma (SMCL)”. Our previously proposed definition of SMCL was as follows: lack of B symptoms, Ki-67 expression less than 30%, low MCL-International Prognostic Index (MIPI) score, normal serum LDH and b2-microglobulin levels, PET/CT with the SUVmax < 6, maximum tumor diameter less than 3 cm, spleen size < 20 cm, with some particular immunophenotype, such as CD5 (−) and CD38 (−), markedly increased CD23 positive lymphocyte proportions (compared to usual negative CD23), kappa light chain restriction (compared to typical lambda light chain restriction), high expression of CD200, without C-myc, NOTCH1/2 and TP53 mutation, nonblastoid/pleomorphic histology, and no tumor growth on reevaluation every 3 months, at least 6 months [16]. In recent years, various techniques, such as polymerase chain reaction (PCR), Sanger sequencing, and next-generation sequencing (NGS) approaches, including whole-exome sequencing (WES), whole-genome sequencing (WGS), and targeted panels, have revealed mutations with prognostic significance in MCL [2]. For example, TP53 mutations are associated with blastoid morphology, and CD200 positivity portends an indolent clinical course [1, 17]. However, there is no systematic analysis representative of the molecular features of indolent MCL. Therefore, in this review, we discuss the molecular characteristics and genomic landscape of indolent MCL to distinguish indolent MCL from classical MCL.

**Table 1:** 2017 WHO classification of mantle cell lymphoma

Mantle cell lymphoma (MCL)	
Classical MCL	Mostly SOX11 ( +)
	Unmutated/minimally mutated IGHV
Indolent leukemic non-nodal MCL^a^	Mostly SOX11 (−)
	Mutated IGHV
In situ mantle cell neoplasia	New name for in situ MCL, reflecting low clinical risk

^a^Indolent leukemic non-nodal MCL with peripheral blood, bone marrow, and sometimes splenic involvement, may become more aggressive

## The difference in the Ki-67 index between indolent MCL and classical MCL

A low Ki-67 index may imply a more indolent form of MCL [18]. Kimura et al. also indicated that Ki-67 expression in small-MCL was lower than that in classical MCL [15]. Ki-67 is a nuclear protein involved in the regulation of cell proliferation [19]. It is not only the most frequently used determinant of cell proliferation in clinical practice but also a predictor of overall survival (OS) [6]. The higher the Ki-67 index, the shorter the time it takes for MCL to transform into blastic and/or pleomorphic MCL. However, its prognostic value in lymphoma is still inconclusive [19]. Some studies have shown that high Ki-67 expression is associated with poo survival rates, while others have shown no strong associations. Vose et al. demonstrated that a high Ki-67 index is associated with MCL subtypes that have a poor prognosis, such as the blastoid variant subtype [18]. Using Ki-67 positivity cutoffs of < 10%, 10–30% and > 30% of cells, Determann et al. demonstrated 3-year OS rates of 93%, 74% and 66% in MCL patients [6]. Moreover, Gallo et al. reported that the Ki-67 index was lower in leukemic non-nodal MCL (average 2%) than in classical MCL (40%) and aggressive MCL (76%), conferring a better prognosis [20]. Therefore, the proliferative activity reflected by the Ki-67 index has become a strong and independent variable for predicting survival in MCL patients. Hoster et al. suggested that the use of the Ki-67 index is superior to cytology and growth pattern as prognostic factors in MCL [21]. Except for MIPI, the Ki-67 index remains the only routinely available independent prognostic factor. However, the Ki-67 index was already accepted and incorporated into the MIPI in 2017 [22]. The modified combination of the Ki-67 index and MIPI showed a refined risk stratification, reflecting their strong complementary prognostic effects. Furthermore, in MCL, the Ki-67 index increases over time, and the only determinant of recurrence risk found was a Ki-67 level of > 30% [23]. In the study of Chakhachiro et al., of 11 patients with a Ki-67 level > 30%, seven experienced disease recurrence within the first 3 years, while only 3 of 16 patients with a Ki-67 level ≤ 30% experienced relapse. However, Medani et al. indicated that it is easier to perform precise counts and accurately evaluate proliferation indices via immunohistochemistry for phosphohistone H3 (PHH3), a reliable mitosis-specific marker in MCL, than via the Ki-67 index [24]. Moreover, Schrader and colleagues found that during early G1 phase, Ki-67 is undetectable, whereas minichromosome maintenance protein 6 (MCM6) is expressed throughout the entire G1 phase. Therefore, high MCM6 expression is a prognostic marker superior to the Ki-67 index in MCL because MCM6 may indicate early G1-phase arrest [25]. Another issue with the Ki-67 index is interobserver variability, making it difficult to determine the best cutoff values across laboratories [6]. Thus, the Ki-67 index plays an important role in the prognosis of MCL and the classification of subtypes [26]. Given that the Ki-67 index of indolent MCL is very low, Ki-67 may be an independent prognostic factor.

## CD200 positivity in indolent MCL

CD200 expression in MCL indicates a unique subgroup. This subgroup is frequently accompanied by IGHV mutations and SOX11 negativity and portends an indolent clinical course [9, 10]. CD200, formerly known as OX-2, is a transmembrane type Ia glycoprotein expressed on thymocytes, activated B cells, T cells, dendritic cells, endothelial cells, and neurons. CD200 transmits inhibitory signals, resulting in the suppression of T-cell-mediated immune activation. CD200 and its ligand CD200R play important roles in the regulation of antitumor activity [27–30]. Recent reports have proven that CD200 immunophenotyping is useful in the differential diagnosis of B-cell neoplasms. CD200 is uncommonly expressed in classical MCL, but it has been noted that CD200 is frequently expressed in indolent MCL. Compared with patients with CD200-negative MCL, those with CD200 + MCL are more likely to have non-nodal leukemic presentation characterized by the loss of lymphadenopathy or extranodal and/or gastrointestinal tract disease [10, 31]. Therefore, CD200 expression is useful for identifying patients with MCL who may have an indolent course [30]. In addition, CD23-positive MCL is more often associated with CD200 positivity and weak SOX11 expression. Recent data have suggested that patients with CD23 + MCL are significantly more likely to have leukemic non-nodal presentation than those with CD23-negative MCL (42% vs 11%) [32]. Kelemen et al. showed that the frequency of bone marrow (BM) and peripheral blood involvement in patients with CD23 + MCL was similar, but extranodal nonmedullary disease was more common. In other words, although patients with CD23-positive MCL have a leukemic presentation similar to CLL, their prognosis is better than that of patients with CD23-negative MCL. In contrast, Saksena and colleagues indicated that patients with CD23 + MCL were more frequently in stage 4 disease with BM involvement and an elevated leukocyte count. In conclusion, indolent MCL is generally CD200 + , CD23 + , and SOX11-negative and has a leukemic presentation with features similar to those of CLL/SLL. In earlier studies, CD200 was proven to be a useful marker for distinguishing CLL from MCL via flow cytometry [27]. However, several recent studies have demonstrated that the IgH-cyclin D1 rearrangement is necessary for the differential diagnosis between MCL and CLL/SLL [30, 33]. CD200 is also a reliable auxiliary marker for classic prognostic factors [29]. The assessment of CD200 is helpful for distinguishing most cases of monoclonal asymptomatic lymphocytosis and cyclin D1–positive (MALD1) indolent MCL from classical MCL to avoid overdiagnosis and unnecessary treatment [17]. Thus, CD200 is frequently expressed in indolent MCL and is associated with CD23 and SOX11.

## Abnormal expression of TP53

Mutations and deletions of the tumor protein p53 (TP53) gene are the most frequent genetic alterations detected in human tumors, although they are rather less common in lymphomas [34]. However, acquisition of the TP53 mutation has been proven to be one of the characteristic markers of MCL. Indeed, in MCL, 26% of cases contain TP53 mutations/deletions [35]. TP53 functions mainly as a transcription factor, phosphorylating at multiple sites, and responds to a large number of cellular stresses, such as cell cycle control, DNA repair, senescence, cell metabolism and apoptosis [36]. The phosphorylation and stabilization of TP53 induced by DNA damage is an obstacle to tumorigenesis. Recent studies have shown the poor prognostic impact of TP53 gene aberrations on MCL patients, including those with the indolent MCL subtype [5, 37, 38]. TP53 mutation and TP53 deletion are both associated with significant reductions in OS in patients with indolent MCL [39]. Conversely, the frequency of TP53 mutations has been reported to be much lower in the indolent variants of MCL [1]. However, TP53 mutations may be clonal or subclonal. The seemingly indolent MCL may contain subclonal TP53 mutations [9]. Several methods can be used to identify TP53 aberrations in clinical samples: (i) a negative feedback mechanism between MDM2 and TP53; (ii) DNA sequencing; (iii) functional assays, especially FASAY (functional analyses of separated alleles in yeast); and (iv) FISH (fluorescence in situ hybridization) [40]. In one study, Streich et al. demonstrated that the majority of TP53 mutations (75%) were associated with deletion of the chromosome arm 17p. Moreover, all TP53-mutated cases show strong TP53 expression by immunohistochemistry [41]. These studies also indicated that TP53 mutations were associated with blastoid morphology and a decreased response to chemotherapy. However, another study showed that the deletion of 17p may not have a prognostic impact [39]. Dong et al. found that TP53 mutations were correlated with a mutated IGHV status and CD38 negativity [42]. In a univariate analysis, TP53 mutations were identified as important predictors of survival, but they were insufficient to be used as independent prognostic factors in patients in the advanced stage. Zlamalikova et al. found 20 cases with loss of the TP53 locus, half of which harbored a concurrent TP53 mutation [34]. Compared to TP53 deletions, TP53 mutations are associated with significantly worse outcomes [5]. Similarly, in chronic lymphocytic leukemia (CLL), > 70% of patients with TP53 deletion also carry TP53 mutation. Therefore, there is a rather weak association between TP53 allelic deletion and TP53 mutation [34]. However, some scholars suggest a lack of prognostic significance for TP53 aberrations in patients with indolent MCL. This may be partly due to the frequent subclonal nature of these mutations in this variant [9]. Eskelund et al. showed the prognostic significance of TP53 mutations but not TP53 deletions. One possible explanation for this difference is that TP53 mutations destroy the function of the entire TP53 protein tetramer, and deletions only reduce the amount of transcription, so they may have little effect on protein function [43]. Furthermore, McCall et al. presented a rare case of CD5-negative non-nodal MCL accompanied by TP53 mutation/17p deletion, but the patient still achieved long progression-free survival (PFS) [44]. Some studies have indicated that the higher the MCL risk group, the higher the percentage of patients with > 50% TP53 expression, and the expression of TP53 is related to the outcome of MCL independent of the MIPI and Ki-67 level [14, 45]. However, some studies have suggested that stratifying patients by proliferation to separate them into low-, intermediate- and high-risk groups is more effective than stratifying them by any single driver mutation [45, 46]. Interestingly, Lin et al. showed no significant difference in prognosis between patients with and without TP53 alterations who received reduced-intensity or nonmyeloablative allogeneic hematopoietic cell transplantation, providing a beneficial treatment modality for these high-risk patients for the first time [47].

Overall, indolent MCL carries almost no TP53 mutation/deletion. However, a small portion of indolent MCL may harbor subclonal TP53 mutations. TP53 mutation and TP53 deletion are both associated with a poor prognosis independent of the MIPI, and TP53 mutations are associated with significantly worse outcomes than TP53 deletions. Moreover, TP53 mutations are correlated with CD38 negativity and hypermutated IGHV.

## The expression level of SOX11

The clinical features of MCL are closely related to the expression level of SOX11. Leukemic mantle cell lymphoma limited to the blood and bone marrow is characterized by a lack of SOX11, mild-moderate lymphocytosis, and interstitial low-level bone marrow involvement [48]. SOX11, a member of the SOXC family, is a single-exon gene and a high-mobility transcription factor [3, 6]. The related transcription factors SOX11, SOX12 and SOX4 compete for the same target genes, and their developmental necessity is evident from single-gene knockout studies indicating that mice without SOX11 and SOX4 expression cannot survive because of heart outflow tract malformations [49]. SOX11 is considered an oncogene that can induce cell proliferation, enforce the expression of PAX5 and inhibit terminal B-cell differentiation into plasma cells via BCL6 and PRDM1 [50]. In addition, SOX11 mediates the expression of platelet-derived growth factor alpha (PDGFA), focal adhesion kinase (FAK) and C-X-C motif chemokine receptor 4 (CXCR4), which promote angiogenesis, metastasis and tumor cell migration, respectively [51]. MRQ-58 has been applied in the study of SOX11 and B cell lymphoma because it has been proven to be the most sensitive marker and does not cross-react with the SOX4 protein, which has high amino acid sequence similarity with SOX11 [52]. SOX11 is not expressed in normal lymphocytes but is widely expressed in MCL [6]. Previous studies have identified several direct targets of SOX11 in MCL, including DBN1, HIG2, SETMAR and WNT signaling molecules. More recently, an elegant integrative analysis of the epigenome in primary MCL demonstrated a distant regulatory element 675 kb downstream from the SOX11 gene that may influence transcriptional activity at the SOX11 promoter [53]. The expression of the SOX11 gene is high in classical MCL and almost absent in indolent MCL [4, 18]. Agata et al. found fewer chromosomal aberrations and more hypermutated immunoglobulin receptor genes in SOX11-negative MCL patients. The SOX11 promoter region was heterogeneously methylated in SOX11-negative primary MCL cases [49]. Moreover, SOX11 is a highly specific marker for both CCND1-positive and CCND1-negative MCL [15]. Variants with a low frequency of SOX11 negativity and IGHV-mutated genes are more indolent and associated with better prognosis. It is useful to confirm SOX11 negativity with hypermutated IGHV to identify a clearly indolent disease [54, 55]. Sílvia et al. demonstrated that SOX11 knockdown reduced engrafted tumor growth in vivo, which is consistent with the indolent clinical course of human SOX11-negative MCL [38]. However, the significance of SOX11 negativity in indolent MCL is still controversial [4]. A retrospective analysis of 186 MCL patients indicated no significant correlation between the absence of SOX11 and the prognosis of MCL [56]. In some studies, patients with SOX11-negative MCL even had a worse prognosis than those with SOX11-positive MCL [39, 57]. There are many factors that could have led to this conclusion: (i) the inclusion of patients with critical SOX11 expression levels, (ii) SOX11-negative cases could correspond to a progressive or transformed stage and generalized lymphadenopathy, (iii) technical difficulties, (iv) the lack of international guidelines for classifying MCL into treatment groups according to clinical behavior (indolent versus classical MCL), (v) the use of heterogeneously treated patients as a basis for the prognostic analysis of SOX11, (vi) potential cross-reactivity of the polyclonal reagents used, (vii) and other confounding factors, such as the presence of TP53 alterations [58]. A highly sensitive and specific in situ hybridization assay for the quantification of SOX11 mRNA in MCL revealed a close correlation between TP53 and negative/low SOX11 expression levels [50, 59]. SOX11-negative cases may represent indolent MCL that obtains TP53 mutations [60]. That is, SOX11-negative MCL initially has an indolent course but becomes aggressive when TP53 mutation is acquired [61]. Consequently, the expression of SOX11 alone should not be used to define aggressiveness. Christian et al. suggested that in both SOX11-negative and SOX11-positive subtypes, the stage of MCL lesions in situ was similar [62]. The expression of SOX11 in “in situ” mantle cell neoplasias suggested that the upregulation of this transcription factor was an early event in MCL [63]. Therefore, SOX11 should not be considered a mere “prognostic parameter” in MCL but rather a marker helping to distinguish the two subsets. However, Nygren et al. showed that a number of indolent MCLs express SOX11 and that in SOX11( +) MCL, indolent disease cannot be ruled out [64]. SOX11 has always been one of the controversial topics of research on indolent MCL. Venera et al. confirmed that SOX11 downregulated hypoxia-inducible gene 2 (HIG-2) at the protein level. HIG-2 knockdown leads to reduced levels of SOX11 [65]. This discovery may provide new clues for the treatment of MCL.

Overall, the expression of the SOX11 gene is almost absent in indolent MCL. SOX11 negativity indicates a better prognosis. However, the significance of SOX11 negativity in indolent MCL is still inconclusive.

## MYC rearrangement or overexpression

Velden and colleagues suggested that B-cell prolymphocytic leukemia is a specific subtype of MCL [66]. Interestingly, patients with prolymphocytic leukemia-like MCL with MYC amplification, no expression of CD38, and loss of TP53 showed prolonged survival outcomes similar to those with indolent leukemic MCL. MYC (8q24) is a critical global transcription factor that regulates 10–15% of all human genes and controls many cellular functions, including the cell cycle, survival, cell growth, apoptosis and metabolism [67, 68]. Contrary to the research of Velden et al., most studies have demonstrated that overexpression/structural changes of MYC in MCL are related to a more aggressive course, a higher MIPI and a worse prognosis [69, 70]. Similar to MYC rearrangements, IGH-BCL2 is also commonly observed in MCLs, and approximately 60% are double-hit lymphomas [67]. MCL with MYC rearrangement is characterized by p53 expression, a high proliferation rate and a complex karyotype [71]. Patients with high MYC or p53 expression have significantly shorter OS and progression-free survival (PFS) [72]. Furthermore, alterations in TP53 and MYC add prognostic information to somatic gene copy number alteration (CNA), enhancing the prognostic correlation of these individual changes observed in previous studies [1]. Choe et al. suggested MYC overexpression to be a predictor of a poor prognosis in MCL [72]. However, Wang and colleagues indicated that MYC rearrangement rather than extra MYC copies is an independent prognostic factor in patients with MCL [67]. Interestingly, there is a close relationship between MYC and miRNAs. MiR-34a was associated with poor outcomes in two independent series of leukemic and nodal MCLs and correlated with high expression of the MYC oncogene [73]. MYC plays an important role in intrinsic ibrutinib resistance in MCL, probably by inhibiting miR16-1 and miR15a, two tumor suppressor miRNAs involved in MCL pathogenesis. The status of MYC could also predict the response to BTKi [67].

Thus, the role of MYC amplification is currently unclear, with this aberrancy reported in studies on both indolent and aggressive MCLs.

## IGHV mutations

As early as 2003, Orchard et al. suggested that mutated immunoglobulin heavy chain variable region (IGHV) genes might help identify indolent MCL [74]. In recent years, some cases of MCL have been characterized by IGHV mutations, and they are truly associated with an indolent disease course [57, 59]. The typical clinical presentation of patients with IGHV mutations comprises leukemic non-nodal CLL-like, including splenomegaly, a Ki-67 proliferation fraction < 10%, a low tumor burden and noncomplex karyotypes [55]. The identified VH rearrangements in indolent MCL were VH1, VH2, VH3 and VH4 [3]. Ferna`ndez et al. compared the disease behavior of indolent MCL with that of classical MCL and showed that 70% of patients with indolent disease carried hypermutated IGHV, and of those mutations, 50% harbored a VH4 mutation [8]. If IGHV genes have been highly mutated, the clinical course is indolent, with involvement of the peripheral blood, and treatment is not necessary [6]. However, the clinical significance of IGHV mutations in MCL is controversial. Some series have shown prolonged patient survival with hypermutations, but most studies have suggested no clear survival benefit [8]. In some instances, the IGHV gene mutation status has been associated with significant reductions in OS [39]. Moreover, the response to chemotherapy and 5-year survival are negatively correlated with the degree of IGHV gene mutation [75].

Thus, most patients with indolent MCL carry IGHV mutations. However, the significance of hypermutated IGHV in patient survival is still conflicting.

## L-MCL16 assay and MCL35 assay

In a recent study, Guillem et al. developed a novel gene expression assay that could identify classical MCL and leukemic non-nodal MCL— the L-MCL16 assay [4]. The L-MCL16 assay includes 16 genes (13 genes that are upregulated in classical MCL and 3 novel genes that are upregulated in non-nodal MCL: CD200, SLAMF1 and BTLA). This novel assay was performed in an independent cohort of 70 leukemic MCL patients and suggested that non-nodal MCL patients predicted by the L-MCL16 assay had significant biological and clinical differences compared to classical MCL patients at diagnosis. Although the L-MCL16 assay recognized three subgroups, high, standard and low proliferation, of classical MCL and leukemic non-nodal MCL, the predictive ability was not well correlated, as previously found in nodal samples. However, their findings emphasized the value of the L-MCL16 assay for the biological determination of indolent MCL. Although the Ki-67 score can be used as a prognostic factor of MCL, Ki-67 staining and interpretation are affected by considerable interlaboratory and interobserver variability [14]. Gene expression analyses are very accurate; however, they are carried out on fresh frozen tissue, which is not easy to obtain for most patients. Therefore, the MCL35 assay, a new identification method, was developed. The MCL35 assay is a proliferation detection method based on the expression of Nanostring-based RNA. It is composed of 35 different genes, 18 of which are housekeeping genes and 17 of which are related to proliferation [76]. The MCL35 assay translated the proliferation characteristics derived from studies on MCL into a test suitable for routinely available formalin-fixed paraffin-embedded (FFPE) biopsies. Scott et al. demonstrated that the MCL35 assay produced gene expression levels of sufficient quality to specify an assay score and risk group in 108 FFPE biopsies from 110 samples (98%). The MCL35 assay, as a continuous score, stratified patients into high-risk, standard-risk and low-risk groups in a training set containing 47 biopsies, with different OS times (medians of 1.1 years, 2.6 years, and 8.6 years, respectively). The MCL35 assay has been shown to have good reproducibility in different centers. Furthermore, the prognostic ability of the assay was validated in younger patients for whom there was a plan to undergo autohematopoietic stem cell transplantation (ASCT). However, Holte et al. suggested that the MCL35 assay cannot separate the standard-risk group from the low-risk group. Furthermore, the MCL35 assay might identify a subgroup with a terrible outcome despite treatment with very active therapy. Moreover, recent studies have suggested that the MCL35 assay is independent of the MIPI but is strongly associated with the Ki-67 index and MCL cytology (classical versus pleomorphic/blastic types). The MCL35 assay was able to identify the three risk groups within the patient subgroup with a Ki-67 index ≥ 30%, and the MCL35 assay subsumed the prognostic power of the Ki-67 PI in pairwise multivariable analysis. Therefore, the MCL35 assay showed stronger prognostic power than the Ki-67 index. However, inversely, some scholars have pointed out that the outcomes of patients stratified according to the Ki-67 score (≥ vs. < 30%) alone is better than the outcomes predicted by the MCL35 assay [77]. Hilka et al. also suggested shortcomings of the MCL35 assay, such as being established only for biopsies from involved lymph nodes with a high tumor load (≥ 60%), poor fixation of the FFPE sample and failure from unknown reasons (10% of MCL specimens). Intriguingly, a recent study showed that the MCL35 assay together with the MIPI and Ki-67 could not predict outcome in nodal samples from patients with indolent MCL who adopted an attitude of waiting.

Therefore, the L-MCL16 assay and MCL35 assay may be used to screen out indolent MCL from all variants of MCL. However, their prognostic value remains unclear.

## P16 aberrations

Deletions of the P16 gene are closely associated with aggressive MCL subtypes, such as the blastoid variant of MCL, with a poor prognosis [18, 78]. P16, also known as INK4a or CDKN2A, is a tumor suppressor gene [79]. The P16 gene is frequently silenced in various types of lymphoma, and this silencing is usually caused by the hypermethylation of CpG islands in its promoter but may also be achieved through the activation of Bmi-1. Streich et al. indicated that 7% of MCL cases in their study harbored codeletions of TP53 and CDKN2A [41]. Of note, the combination of both aberrations represents significantly more adverse prognostic effects than the isolated aberration of either gene [5]. Moreover, the prognostic effects of p16 and TP53 deletions were not related to the proliferation marker Ki-67 [80]. Importantly, patients with concurrent TP53 mutation/deletion and P16 deletion might benefit from innovative treatments, such as Bruton’s tyrosine kinase inhibitors (BTKi). Thus, the expression level of P16 is normal or higher in indolent MCL.

## ATM defects

The frequency of defects in ataxia telangiectasia mutated (ATM) gene has been reflected to be much lower in the indolent variants of MCL [9]. The ATM gene encodes a 370-kDa ATM protein, which is a member of the PI-3 protein kinase family and is essential for DNA processing, the cell cycle and telomere length regulation [81]. Notably, 11q deletion and/or ATM mutation, in cooperation with cyclin D1, consistently represent the most frequent oncogenic event in MCL [82]. This response is closely related to the activation (stabilization) of TP53 mediated by ATM. Indeed, in MCL, 26% of cases contain TP53 mutation/deletion, 56% contain ATM alteration and 10% contain both genetic changes [35]. Mareckova and colleagues revealed the mutual exclusivity of ATM and TP53 mutations in MCL [82]. Due to limited information on the clinical impact of ATM mutations on MCL and although ATM has been repeatedly proven to be the most commonly mutated gene, followed by CDKN2A and TP53, the latest publications consistently show no statistically significant difference in the OS of patients with wild-type vs. mutated ATM, which is in sharp contrast to TP53 defects [3, 83]. Nevertheless, Delfau et al. reported that somatic gene copy number alterations in MYC, CDK2, ATM, MDM2 and CDK4 had no prognostic value [80]. Of note, however, non-nodal leukemic MCLs with aberrations in ATM are more aggressive [61]. In the report of Bea et al., ATM mutations were found in more than half of tumors with SOX11 positivity but not in those with SOX11 negativity [84]. ATM was present at similar allelic frequencies in the two subclones of different cases, indicating that it represents early events. Furthermore, interestingly, the loss of ATM may actually contribute to the radiosensitivity of MCL cells [85].

The frequency of defects in ATM is much lower in indolent MCL. Furthermore, indolent MCLs with aberrations in ATM are more aggressive. However, some recent studies have suggested that ATM has little prognostic value.

## NOTCH1/2 mutations

The frequency of mutations in the NOTCH1 gene has been reported to be much lower in the indolent variants of MCL [9]. Other studies have indicated that indolent MCL lacks NOTCH1/2, MYC and TP53 mutations [16]. In other words, NOTCH1 and/or NOTCH2 mutations have been related to poor outcomes [43]. Jain et al. suggested that patients with variants of aggressive histology MCL (AH-MCL) frequently exhibited CCND1, NOTCH1, and SMARCA4 (SWI/SNF) gene mutations [86]. Moreover, the presence of NOTCH2, UBR5, and NOTCH3 mutations was exclusive to AH-MCL. Zhou and colleagues believe that MYC rearrangement together with NOTCH2 mutation contributes to the aggressive subtype switch from CD19 + CD10– cells to CD19 + CD10 + cells [87]. Interestingly, NOTCH2 mutation is a substitute for NOTCH1 mutation [84]. However, NOTCH1 was not identified as an independent prognostic factor in a multivariate model with TP53 mutations [46]. In conclusion, 9.5% of MCL patients present NOTCH1/2 mutations, which can be used to identify a subset of tumors with more aggressive biological and clinical features, including those with blastoid/pleomorphic morphology. Indolent MCLs express no or low NOTCH1/2.

## Several genes that have been valued in recent years

**Fig. 1:**
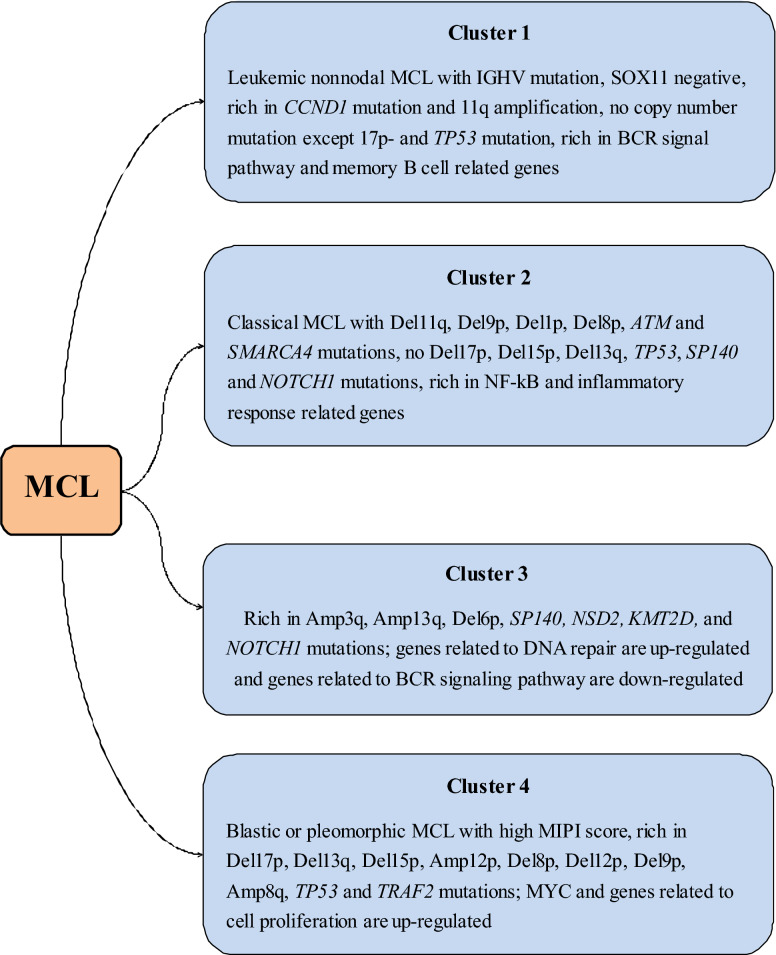
New classification of MCL

Compared to those with a low Ki-67 level, AH-MCL patients with Ki-67 ≥ 50% have exclusive mutations in TP53, RANBP2, SMARCA4, KMT2C, SPEN, and NSD2 (WHSC1) [86, 88]. The study group of Sakhdari et al. included twenty-one (81%) patients with nodal MCLs and 5 (19%) patients with leukemic variant MCLs. In their study, TP53, CARD11, ATM, FBXW7, SPEN, NOTCH1 and BIRC3 were mutated to varying degrees, and most mutations were clonal in nature [9]. Among these mutated genes, SMARCA4 and BCL2 were altered only after progression, while TP53, KMT2D (MLL2), CDKN2A, CCND1, CELSR3, NOTCH2 and ATM were altered 2–4 times more frequently after progression [89]. Zhao et al. demonstrated that in the progression of MCL, there was clonal evolution of novel SMARCA4 and KMT2C/D mutations. However, Jain et al. did not observe BIRC3, MAP3K14 and TRAF2 mutations at any time point in their experiment [90]. Yang and colleagues suggested that after the MIPI and MIPI-c score, TP53 and WHSC1 mutations were the most significant prognostic factors in MCL, and all patients benefited from WHSC1 and TP53 mutations at diagnosis [91]. KMT2D mutations are associated with an increased risk of progression and death [92]. By adding KMT2D mutations and TP53 disruption to the MIPI-c backbone, Ferrero et al. derived a new prognostic indicator, the “MIPI-genetic”. The “MIPI-g” improved the discrimination ability of the model, defining low-risk, intermediate-risk and high-risk patients (4-year PFS: 72%, 42.2%, and 11.5%, respectively; 4-year OS: 94.5%, 65.8%, and 44.9%, respectively). They also demonstrated that among patients with TP53 disruption, those with KMT2D mutations had a poor outcome. Some researchers described the genetic spectrum of 134 MCL patients, and they suggested that ATM and KMT2D mutations were more frequent in patients without IGHV mutations. Abnormal SP140 expression and PCDH10 mutation independently imply shorter PFS and OS. Del 9p and/or Del 9q, the most common variants, were found in 40% of the 134 patients and were significantly associated with a poor prognosis. In contrast, abnormalities in the Amp 11q gene are associated with prolonged PFS and OS. The researchers further divided the samples into four groups: no chr9 deletion, major Del 9q, major Del 9p and massive chr9 deletion. They found that the prognosis of the group without chr9 deletion was significantly better than that of the other three groups, suggesting that tumor suppressor genes may be located on the part of chr9 that is deleted [93]. Recently, a new classification of MCL that can predict prognosis was proposed (Fig. 1). The altered splicing of HNRNPH1 was related to inferior outcomes in MCL patients [46]. All patients with WHSC1, MLL2 and UBR5 mutations responded to therapy. Conversely, alterations in NOTCH1, CCND1 and SMARCA4 occurred only in nonresponders [94]. The deletion of SMARCA4 results in transcriptional changes that enable cells to survive a therapeutic challenge, and this is reflected in the fact that SMARCA4 mutations are enriched in patients with primary progression. Interestingly, mutations in CARD11 and BIRC3 were previously associated with ibrutinib resistance and regulation of the activation of NF-κB, but this did not rule out the response to the combination of ibrutinib and venetoclax. Overexpression of CARD11 mutants was demonstrated to endow resistance to the NF-κB inhibitor lenalidomide and the BCR inhibitor ibrutinib [95]. Moreover, mutations in the SWI–SNF chromatin-remodeling complex confer resistance to venetoclax and ibrutinib. Last, in future analyses, changes in the mutational status from baseline samples to samples at disease progression and current mutations of interest in MCL should be taken into account [2].

In conclusion, the frequency of mutations in SPEN, SMARCA4 (SWI/SNF), RANBP2, KMT2C, NSD2 (WHSC1), Del 9p and/or Del 9q, CARD11, FBXW7, SP140, BIRC3, KMT2D (MLL2), PCDH10, CELSR3 and HNRNPH1 is much lower in indolent MCL. Conversely, Amp 11q gene mutations without chr9 deletion have a better prognosis.

## Genes that lack data

Indolent MCL is characterized by kappa light chain restriction. In contrast, a more typical lambda light chain restriction is presented in cMCL [48, 96]. The incidence of frameshift Bax mutations is low in indolent and mantle cell lymphomas, which indicates that microsatellite instability (MSI) is not a feature of the pathogenesis of these lymphomas [97]. Petrakis et al. showed a close positive correlation between the expression of CD34 and SOX11 [59]. However, we still cannot draw a conclusion on the correlation between SOX11 expression and CD83 expression in MCL. The levels of cell membrane CD83 (mCD83) in MCL patients are significantly elevated. The expression of mCD83 is mainly limited to lymphocytes, neutrophils and activated DCs. The CD83 promoter contains NF-κB-binding sites. CD83 expression is correlated with canonical NF-κB activation in MCL [98, 99]. Moreover, indolent MCL shows low or no expression of genes in the high-mobility group (HMG) [100]. Importantly, poly(ADP-ribose) polymerase 1 (PARP1) protein expression is related to a progressive course of indolent MCL and shortened OS. Gallo and colleagues indicated that PARP1 should be included in initial diagnostic studies as a negative predictor [20]. PARP1 is a nuclear protein involved in DNA repair and the maintenance of genomic stability and is overexpressed in a number of aggressive cancers. As reported, through caspase-9–3-7-PARP signaling, SOX11 silencing promotes proliferation and inhibits the apoptosis of MCL cells and desensitizes MCL cells to bortezomib [101]. At diagnosis, the peripheral blood absolute monocyte count (AMC) is an independent prognostic factor for OS in MCL patients, suggesting its ability to predict outcomes in addition to the MIPI [102]. Jain et al. pointed out that the downregulation of BACH2 was related to an increase in proliferation, and patients with cluster 1 methylation patterns with somatic mutations had less aggressive disease [103]. Activation of the Akt pathway has been found in most blastoid MCLs, and the loss of PTEN promotes this process. Additionally, antiapoptotic Mcl-1 overexpression is significantly related to blastoid MCL and TP53 mutations.

Indolent MCL is characterized by kappa light chain restriction, a low incidence of frameshift Bax mutations, low expression of CD34, low or no expression of HMG, low PARP1 protein content, upregulation of BACH2, inhibition of the Akt pathway and low expression of Mcl-1.

## The expression of microRNAs in indolent MCL

Javeed et al. suggested that a set of stroma-associated microRNAs might define a more indolent group of MCL [104]. MicroRNAs (miRNAs) are noncoding single-stranded RNA molecules composed of 21–23 nucleotides that bind to the 3′-untranslated region (UTR) of the target gene messenger RNA (mRNA) [105, 106]. MiRNAs do not encode proteins, but they go through the process from a primary transcript to a precursor miRNA and then to a mature miRNA [107]. MiRNAs are involved in the posttranscriptional regulation of gene expression and important biological processes, including oncogenesis [108]. Studies have shown that miRNAs are frequently deregulated in a variety of human malignancies [107]. Therefore, miRNA expression may predict outcomes in both solid tumors and hematologic malignancies. Previously, the abnormal expression of miRNAs was frequently observed in various types of lymphomas, suggesting their potential benefits in clinical diagnosis [109]. Zhao et al. demonstrated that patients with significantly downregulated miR-29 had short survival and that downregulation of miR-29 together with cyclin D1 had a synergistic effect in the pathogenesis of MCL [107]. Additionally, Navarro et al. suggested that 7 miRNAs showed prognostic significance independent of the IGHV status and SOX11 expression, and among the top 3 were miR-708 and miR-455-5p/3p [73]. Thus, miR-708 and miR-455-5p/3p may well distinguish between IGHV mutated/wild-type and SOX11 positivity/negativity in MCL. This study also suggested that the expression level of miR-181a/c had an effect on leukemic MCL, mainly by downregulating the expression of ATM, and the CD40 signaling pathway was identified as an important target for the differentially expressed miRNAs between unmutated MCL and mutated MCL. Goswami et al. demonstrated that overexpression of miR-20b was associated with a worse prognosis and high proliferation in MCL, as well as low expression of miR-29. Here, compared with miR-127-3p, the expression of miR-615-3p could better distinguish the different prognostic groups in MCL [108]. MiR-223 is a miRNA that has been well studied. MiR-223 expression is also decreased in unmutated IGVH MCLs, and these types of lymphoma have an aggressive clinical course [110]. Overexpression of miR-223 decreases the viability and proliferation of MCL cells and promotes apoptosis, which may be due to the downregulation of SOX11 mediated by miR-223 [106]. A database search showed that SOX11 was the hypothetical target for miR-223. Luciferase reporter assays confirmed that posttranscriptional miR-223 suppressed the 3′-untranslated region of wild-type SOX11 but did not inhibit that of mutated SOX11. Therefore, these studies not only suggest that miR-223 is highly suppressed in MCL patients and associated with high-risk clinical features but also prove the tumorigenic role of SOX11 [63]. MiR-132-3p is very similar to miR-223. The higher expression of miR-132-3p predicts prolonged survival in MCL patients, and its direct target is SOX11 [109]. Husby et al. combined the expression levels of miR-18b with new biological MCL International Prognostic Index (MIPI-B) data for the first time. Overexpression of miR-18b significantly downregulated MCL cell proliferation [111]. The study also suggested a number of prognostic miRNAs, including the entire miR-17 ~ 92a cluster [112].

In conclusion, the expression level of miR-20b in indolent MCL is lower than that in classical MCL. MiR-127-3p and miR-615-3p are related to the OS of patients with indolent MCL. Moreover, miR-29, miR-708, miR-455-5p/3p, miR-223, miR-132-3P and miR-18b are highly expressed in indolent MCL.

## Conclusions

MCL is one of the most aggressive lymphoid tumors, and the toxicity of chemotherapeutic drugs is unfavorable to every patient. Indolent MCL is a subtype of mantle cell lymphoma. Indolent MCL has a better prognosis than classical MCL. However, the process of “wait and watch” puts these patients at the potential risk of evolution to classical and aggressive MCL [16]. Therefore, a clear understanding of the molecular underpinnings of indolent MCL is necessary.

**Fig. 2:**
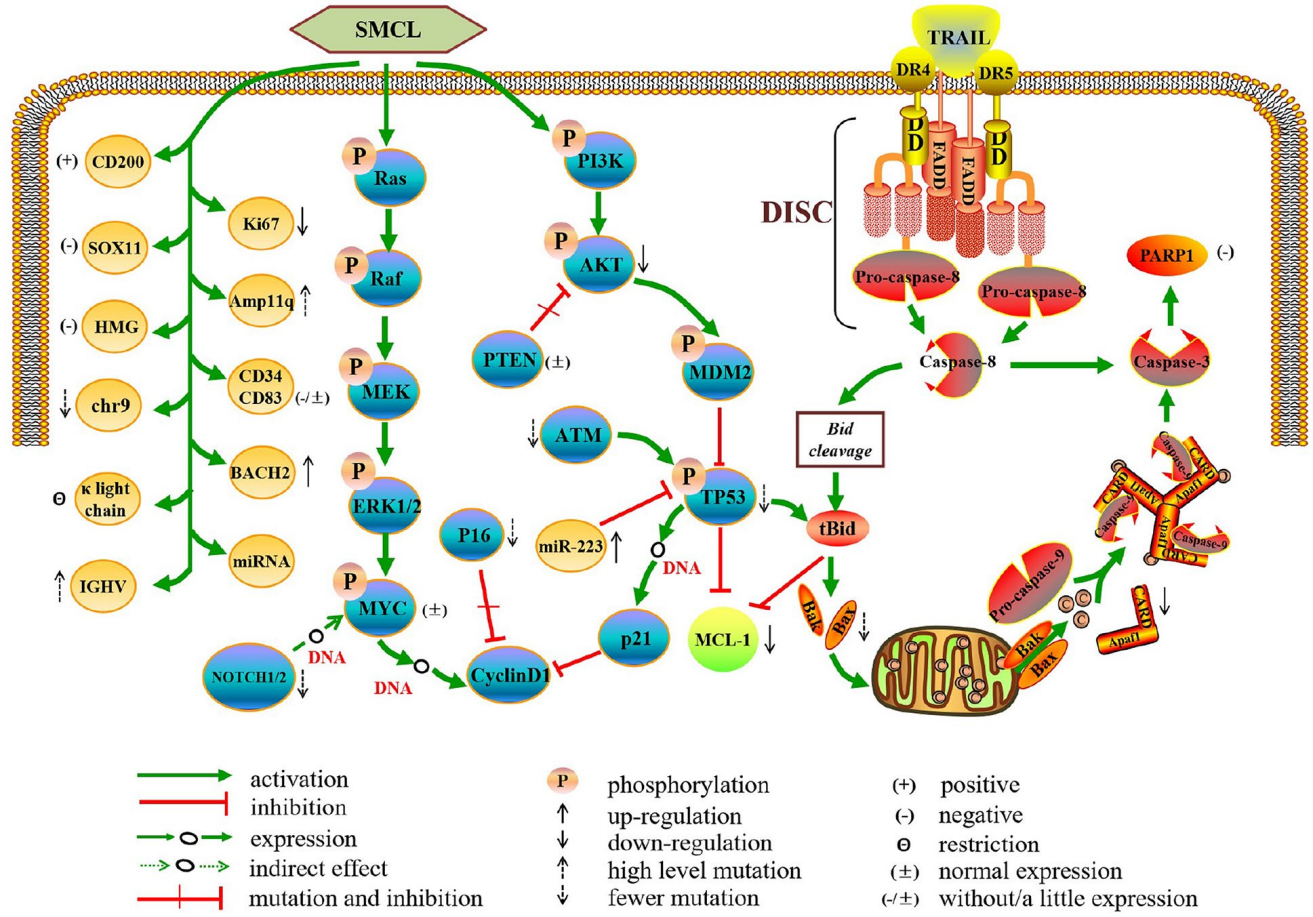
Molecular network of indolent MCL

In this review, we summarize and analyze the common molecular characteristics of indolent MCL described in previous studies (Fig. 2). Furthermore, we focus on several genes that have been valued in recent years and some genes that lack data (Table 2). We firmly believe that a more in-depth study of the molecular characteristics may distinguish indolent MCL from classical MCL. If we can clearly distinguish indolent MCL from classical MCL, indolent MCL will not be overtreated, whereas the treatment of classical MCL will not be delayed.

**Table 2:** Molecular markers of indolent MCL

Ki-67 index ≤ 30%
CD200 positivity
No *TP53* mutation/deletion
Lack of SOX11
Without rearrangement and overexpression of MYC
IGHV mutations
L-MCL16 assay and MCL35 assay
Unmutated *P16* status
Without/minimal defects of ATM
No *NOTCH1/2* mutation
Amp 11q gene mutation
No chr9 deletion
Several genes that have been valued in recent years (all of them are weakly expressed)
* SPEN*
* SMARCA4 (SWI/SNF)*
* RANBP2*
* KMT2C*
* NSD2 (WHSC1)*
* CARD11*
* FBXW7*
* BIRC3*
* KMT2D (MLL2)*
* CELSR3*
* TRAF2*
* MAP3K14*
* HNRNPH1*
Del 9p and /or Del 9q
* SP140*
* PCDH10*
Genes that lack data
Kappa light chain restriction
Almost no incidence of frameshift Bax mutations
CD34 (−/ ±), CD83(−/ ±)
No expression of HMG
PARP1 silencing
Up-regulation of BACH2
Cluster 1 methylation pattern with somatic mutation
Inhibition of Akt pathway
No loss of PTEN
Without overexpression of Mcl-1
MicroRNAs upregulation/downregulation

## Data Availability

Data sharing is not applicable to this article as no datasets were generated or analyzed during the current study.

## Abbreviations

AMC: Absolute monocyte count
ASCT: Auto-hematopoietic stem cell transplantation
ATM: Ataxia telangiectasia mutated
BM: Bone marrow
BTKi: Bruton’s tyrosine kinase inhibitors
CLL: Chronic lymphocytic leukemia
FASAY: Functional analyses of separated alleles in yeast
FFPE: Formalin-fixed paraffin-embedded
FISH: Fluorescence in situ hybridization
HIG-2: Hypoxia-inducible gene 2
IGHV: Immunoglobulin heavy chain variable region
LDH: Lactate dehydrogenase
MCL: Mantle cell lymphoma
MCM6: Minichromosome maintenance protein 6
MIPI: MCL-International Prognostic Index
MSI: Microsatellite instability
NF-κB: Nuclear factor kappa-B
OS: Overall survival
PARP1: Poly (ADP-ribose) polymerase 1
PET/CT: Positron Emission Tomography/computerized tomography
PFS: Progression-free survival
PHH3: Phosphohistone H3
SMCL: Smoldering mantle cell lymphoma
SUV: Standard Uptake Value
TP53: Tumor protein p53
UTR: 3’-Untranslated region
